# A 3D Printed Human Ear Model for Standardized Testing of Hearing Protection Devices to Blast Exposure

**DOI:** 10.1097/ONO.0000000000000010

**Published:** 2022-05-26

**Authors:** Marcus A. Brown, Shangyuan Jiang, Rong Z. Gan

**Affiliations:** 1 Stephenson School of Biomedical Engineering, University of Oklahoma, Norman, OK; 2 School of Aerospace and Mechanical Engineering, University of Oklahoma, Norman, OK.

**Keywords:** 3D printing, Blast overpressure, Ear, Hearing protection devices, Human temporal bone

## Abstract

**Hypothesis:**

A 3D printed human temporal bone (TB) that is anatomically accurate would cost-effectively reproduce the responses observed in blast testing of human cadaveric TBs with and without passive hearing protection devices (HPDs).

**Background:**

HPDs have become critical personal protection equipment against auditory damage for service members. Acoustic test fixtures and human TBs have been used to test and develop HPDs; however, the lack of a cost-effective, standardized model impedes the improvement of HPDs.

**Methods:**

In this study, the 3D printed TB model was printed with flexible and rigid polymers and consisted of the ear canal, tympanic membrane (TM), ossicular chain, middle ear suspensory ligaments/muscle tendons, and middle ear cavity. The TM movement under acoustic stimulation was measured with laser Doppler vibrometry. The TB model was then exposed to blasts with or without HPDs and pressures at the ear canal entrance (P0) and near the TM in the ear canal (P1) were recorded. All results were compared with that measured in human TBs.

**Results:**

Results indicated that in the 3D printed TB, the attenuated peak pressures at P1 induced by HPDs ranged from 0.92 to 1.06 psi (170–171 dB) with blast peak pressures of 5.62–6.54 psi (186–187 dB) at P0, and measured results were within the mean and SD of published data. Vibrometry measurements also followed a similar trend as the published results.

**Conclusions:**

The 3D printed TB model accurately evaluated passive HPDs’ protective function during blast and the potential for use as a model for acoustic transmission was investigated.

Noisy work environments and blast overpressure exposure have caused hearing loss to be a prominent disability among veterans (1,2). Blast-related ear injuries, such as tympanic membrane (TM) rupture, can result in hearing impairments (3–5). Hearing protection devices (HPDs) are necessary for preventing hearing loss while deployed in hazardous environments, and even though various HPDs are widely available, some troops feel HPDs reduce situational awareness (1,6). Advanced HPDs have been developed to increase perceived situational awareness and improve HPD compliance; however, recent studies indicate the need for further HPD improvement with reliable and efficient testing methodologies (6–8).

Previous studies utilized different computational and experimental methods to investigate the efficacy of HPDs when exposed to noise impulses or blast (6,7,9). Utilizing HPDs did attenuate intense impulse noises during human experiments, but early indications of hearing loss were still found emphasizing that assessment of numerous HPD designs in humans would be unethical and cause long-term harm (6,8). Human cadaveric temporal bones (TBs) have been used to investigate the protective function of different earplugs (9); however, cadaveric TBs rely on donor availability and the ear canal size variation add variability to the results (9,10). Acoustic test fixtures have been utilized to test insertion loss of HPDs during exposure to impulse noises (7,11). While Murphy et al (7) did obtain consistent results with an acoustic test fixture, the sound pressure levels (SPLs) used were below that observed in blast exposures where pressure levels could damage the expensive test fixture. Computational models offer a cost-effective and time-efficient method of testing HPDs in high-pressure environments, and Gan et al (9) were able to simulate blast exposure to their ear model with and without HPDs. While the model provided insight into the ear’s response to blast overpressure, the finite element model’s results did exhibit some discrepancies when compared to the experimental results demonstrating that physical models for testing HPDs are essential until computational models improve.

Three-dimensional printing technology provides an intriguing tool for customized solutions for otolaryngology applications (12–14). Three-dimensional printed auricular prostheses and ossicular chain prostheses were used to improve the cosmetic outcome and mechanical function, respectively, in patients, and TM grafts have been developed for restoring the TM to its original structure after tympanoplasty (13–16). Three-dimensional printed models have the possibility to mimic the ear’s function for mastoid surgery (17,18); however, 3D printing has not been utilized to create models for developing HPDs. A model that takes advantage of 3D printing’s benefits could vastly improve the evaluation of HPDs.

This article reports the development of a 3D printed ear model or TB that anatomically and mechanically represented the outer and middle ear for effective evaluation of passive HPDs during blast exposure. It utilized flexible and rigid materials to print the ear canal, TM, middle ear ossicles with suspensory ligaments, and middle ear cavity. Experiments exposed the 3D printed TB to blast overpressures with and without HPDs, and results were compared with similar experiments using cadaveric TBs for validation. These experiments monitored pressure at the ear canal entrance and near the TM. The TM displacement was measured during acoustic simulation and compared to published results to investigate its acoustic test module potential. This novel 3D printed TB was developed with the aim to provide a standardized testing model for the efficient, cost-effective, and repeatable evaluation of HPDs’ performance under blast conditions in hopes to hasten the improvement of HPDs.

## MATERIAL AND METHODS

### Design and Fabrication of 3D Printed TB

**FIG. 1.**
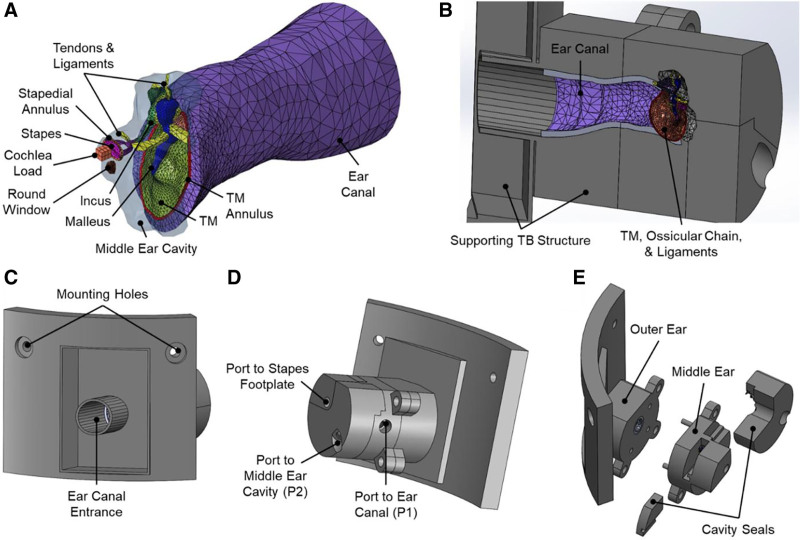
Computer models used for 3D printing, illustrating the finite element model of the ear and 3D printed TB. *A*, Finite element model of the human ear with the ear canal, middle ear, and cochlear load. *B*, Sectional view of the assembled CAD model showing the enclosed ear canal and middle ear tissues. *C*, Lateral view of the CAD model of the 3D printed TB highlighting the ear canal entrance. The model’s shape was designed to mount into the head block for blast exposure tests. *D*, Medial view of the CAD model of the 3D printed TB. The pressure ports to the ear canal near the TM (P1) and middle ear cavity (P2) are shown where pressure sensors are threaded for measurements. A port for access to the stapes footplate for future studies is also shown. *E*, Exploded view of the 3D printed TB’s CAD model showing the parts to be printed separately: outer ear, middle ear, and cavity seals. CAD indicates computer-aided design; TB, temporal bone; TM, tympanic membrane.

The 3D printed TB was designed from an finite element model originally developed by Gan et al (19) using histological cross-sectional images of a 55-year-old human male TB (left ear). The finite element and 3D printed TB model consisted of the structural components of the human outer and middle ear including the ear canal, TM, ossicular chain, middle ear suspensory ligaments/muscle tendons, and middle ear cavity (Fig. 1A, B). An assembled computer-aided design model for 3D printing of TB is shown in Figure 1C–E. The outer ear portion contained most of the ear canal (Fig. 1C) and was designed to attach to the “head block” used in blast exposure tests as described by Gan et al (9), and the middle ear portion contained the middle ear tissues as well as ports for measuring air pressure near the TM (P1) and in the middle ear cavity (P2) and access to the stapes footplate (Fig. 1D).

The model was printed using an Objet350 Connex3 3D printer (Stratasys, Eden Prairie, MN), which can print materials with varying mechanical properties. Flexible materials were used to print the soft tissues (TM, ligaments, tendons, and canal skin) and rigid materials were used to print the hard tissues (ossicles, manubrium, and TB structures). Materials were chosen by their closeness to their respective part’s published mechanical properties (see Table S1, Supplemental Digital Content 1, http://links.lww.com/ONO/A3, for the material properties). Limited information was available for the 3D printer materials’ composition and mechanical properties due to Stratasys’ materials being proprietary (20,21).

### Three-Dimensional Printed TB and Human Cadaveric TB Blast Exposure Tests

**FIG. 2.**
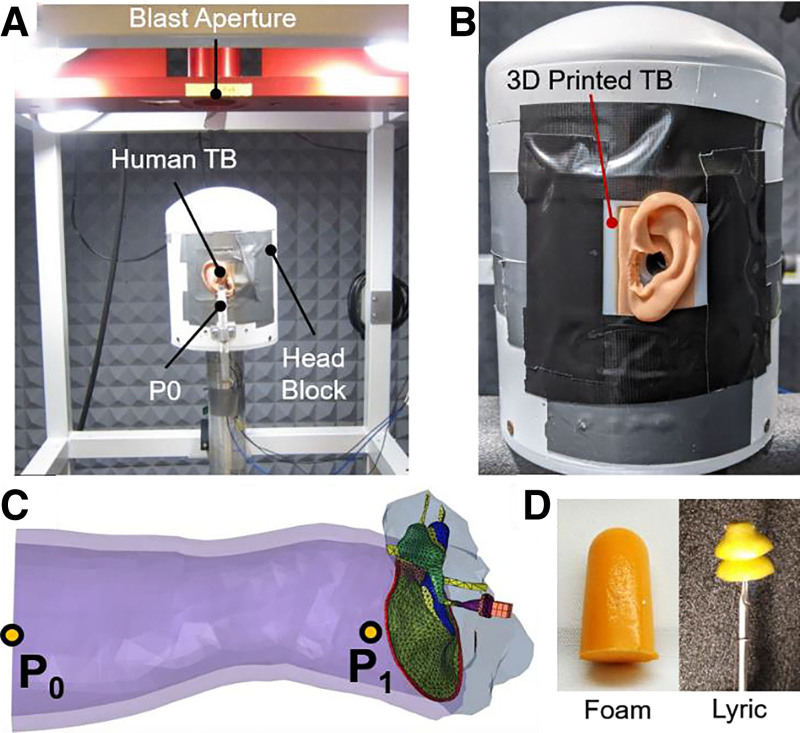
Experimental blast setup and pressure sensor placements used to measure blast wave transmission in ear models. *A*, Image of the blast test setup within an anechoic chamber where the “head block” sits below the blast aperture. A human cadaveric TB is mounted to the head block and the pressure sensor P0 is mounted outside of the ear canal entrance. *B*, Image of the 3D printed TB mounted to the head block. *C*, The finite element model highlighting where the pressures were monitored during blast exposure: at the entrance of the ear canal, P0, and in the ear canal near the TM, P1. *D*, Images of the foam earplug (left) and Lyric earplug with a stick holder (right) used in blast exposure tests. TB indicates temporal bone; TM, tympanic membrane.

Blast exposure tests in 3D printed and human cadaver TBs were performed similar to HPD characterizations performed by Gan et al (9). Briefly, the fresh human TBs were supplied from Science Care, Inc., a certified human tissue supplier for military health research. The study protocol was approved by the Office of Research Protections, US Army Medical Research and Material Command. The 3D printed or human cadaveric TBs were exposed to blast overpressures in an anechoic chamber while fixed to the “head block” under a blast apparatus designed for open-field blasts (Fig. 2A, B). Exploding a polycarbonate film (McMaster-Carr, Atlanta, GA) with nitrogen gas at the blast aperture (Fig. 2A) produced blast overpressure in the range of 6–8 psi. Figure 2C illustrates where pressures were monitored: P0, entrance of the ear canal, and P1, in the ear canal near the TM. P0 pressure sensor (Model 102B16; PCB Piezotronics, Depew, NY) was mounted 1 cm lateral to the entrance of the ear canal (Fig. 2A), and P1 pressure sensor (Model 105C002; PCB Piezotronics, Depew, NY) was implanted into an opening near the TM. Pressure sensor readings were recorded with a cDAQ 7194, A/D converter 9215 data acquisition system (National Instruments Inc., Austin, TX) using LabVIEW Signal Express software (National Instruments Inc., Austin, TX).

Blast tests were performed with and without one of the 2 earplugs inserted into the ear canal of the TBs. A 3M foam earplug (3M Co., St. Paul, MN) was selected for its common use in industry and published literature (7,9,10), and the Lyric hearing aid (Phonak, LLC, Warrenville, IL) was included due to its availability during a collaborative research effort with Walter Reed National Medical Center (Fig. 2D left and Fig. 2D right, respectively). The Lyric hearing aid (or earplug) is a deep insertion hearing aid that is installed 4 mm away from the TM and has the potential to offer hearing protection (9,22). This was installed without the battery in both the human and 3D printed TBs to perform as a passive HPD. Blast exposure to TB models was performed first without earplugs then exposed with an earplug fitted. Cadaveric TB samples were fitted with either the foam (n = 13) or Lyric (n = 6) earplugs, while the 3D printed TB was tested with both (n = 4 for foam and n = 3 for Lyric earplugs).

### Three-Dimensional Printed TB Validation and HPDs Evaluation

To investigate the 3D printed TB for sound transmission through the ear, movement of the TM was measured using laser Doppler vibrometry (LDV) under acoustic stimulation, and results were compared with published human TM’s displacement (23). The LDV measurements performed in this study were similar to those performed by Gan et al (24); however, only TM velocity was recorded. Sound was delivered near the TM from a speaker (Model MF1; TDT, Alachua, FL) through a 1 mm inner diameter tube controlled by a dynamic signal analyzer (HP 35670A; Palo Alto, CA) and a power amplifier (B&K 2718; Norcross, GA). The system’s output SPL was monitored with a probe-tipped microphone (ER-7C; Etymotic Research, Elk Grove Village, IL) 2 mm away from the TM, which allowed the system to generate and maintain a pure tone of 90 dB SPL from 200 to 10,000 Hz. The TM movement was measured by the LDV (Polytec CLV 2534; Irvine, CA) with the laser focusing on a piece of reflective tape attached to the TM’s umbo and recorded by the dynamic signal analyzer.

Comparison between P1 pressure waveforms from the human and 3D printed TBs were used as validation for the TB model as an effective HPD test standard. Measures used to compare P1’s intensity and waveform were the peak pressure level, P1:P0 peak pressure ratio, and A-duration (the measure of time the positive portion of the peak pressure is sustained). In addition, blast attenuation by the earplugs was compared between the 3D printed and human TBs. The percent error among recorded data was used to evaluate the accuracy of the 3D printed TB to the human TB. The human TB blast test data used for comparisons were previously published in Gan et al (9), where blast tests were performed on 13 TBs (age 74.5 ± 7.6) with foam earplugs and 6 TBs (age 79.7 ± 5.2) with Lyric earplugs.

## RESULTS

**FIG. 3.**
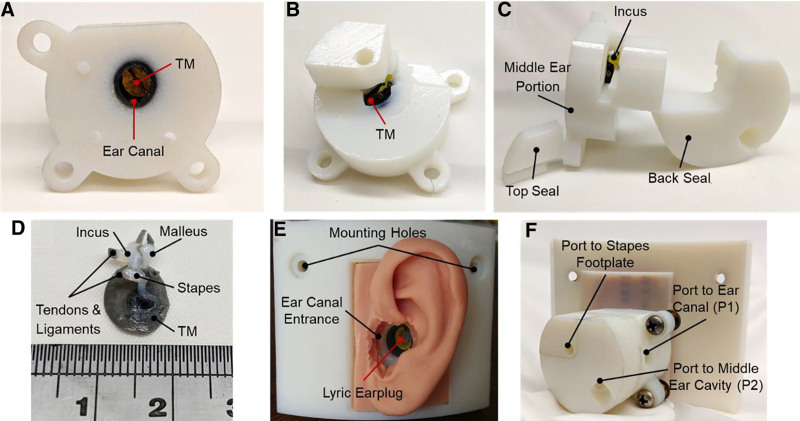
Images of a finished print of the 3D printed TB’s unassembled middle ear portion. *A*, Lateral view of the middle ear portion showing the ear canal and TM within. *B*, Medial view of the middle ear portion of the 3D printed TB showing the opening used for cleaning. *C*, Posterior view of the 3D printed TB’s middle portion with the seals. *D*, A 3D print of the ossicles, TM, and ligaments and tendons from within the middle ear cavity of the 3D printed TB. The ossicles were imaged without the surrounding material and with a scale to highlight the detail and size of the middle ear tissues. *E*, Lateral view of the assembled 3D printed TB with a Lyric earplug inserted into the ear canal. A silicone mold of the pinna was adhered to the 3D printed TB to simulate the outer ear. *F*, Medial view of the assembled 3D printed TB showing the ports to measure the pressures at the TM and in the middle ear cavity and to access the stapes footplate. TB indicates temporal bone; TM, tympanic membrane.

A finished print of the 3D printed TB’s unassembled middle ear can be seen in Figure 3A–D, where rigid materials were printed in either white or yellow (hard tissues) and flexible materials were printed in shades of gray (soft tissues), and an assembled model in Figure 3E, F. The lateral surface of the TM can be seen through the ear canal in Figure 3A, while Figure 3B shows the medial-inferior side of the TM within the middle ear cavity. The ossicular chain was freely suspended after support material removal. The top and back seals (Fig. 3C) were affixed to the middle ear portion to form the middle ear cavity. For clarity, the TM, ossicular chain and their attached ligaments and tendons were 3D printed separately at 1:1 scale (Fig. 3D) and retained its shape throughout the 3D printed TB’s fabrication. The model was designed to mount in our current head block and allow space to adhere a silicone pinna (Fig. 3E). Figure 3E demonstrates how the Lyric earplug would be fitted into the ear canal. Figure 3F highlights the ports to the ear canal (P1), middle ear cavity (P2), and stapes footplate. The port to the stapes footplate was designed to allow the application of a cochlear pressure to the stapes footplate in future applications.

**FIG. 4.**
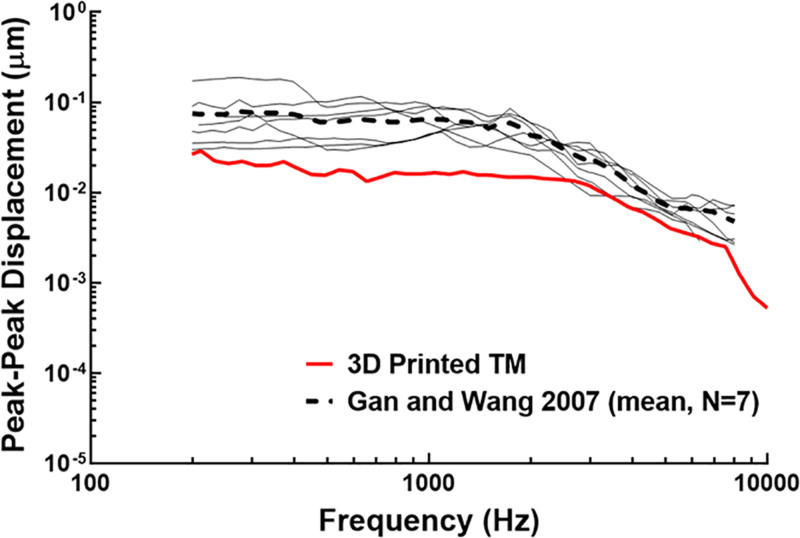
Plot of the peak-to-peak displacement of the TM over the frequency range of 200–10,000 Hz for the 3D printed TB’s TM (red line) and 7 human TB samples (solid black lines) from Gan and Wang (23). The average of the 7 samples is plotted in the black dotted line. TB indicates temporal bone; TM, tympanic membrane.

Figure 4 displays the peak-to-peak displacement of the 3D printed TB’s TM and compares it to data published by Gan and Wang (23). The human TB data were recorded using LDV in 7 human cadaveric TBs with average shown in Figure 4. While the displacement of the 3D printed TM follows a similar trend as the human TM, the displacement of the 3D printed TM was substantially lower than the human’s at frequencies lower than 3 kHz. The greatest discrepancy between the human TB average and 3D printed TM occurred at 275 Hz with a difference of 0.058 µm (72.5% error, 0.080 and 0.022 µm for human TM average and 3D printed TM, respectively), and the closest values occurred at 5248 Hz with a difference of 0.003 µm (41.1% error). It is important to note that the 3D printed TB was designed to test HPDs’ effectiveness in protecting the ear during blast exposure in this study, and future iterations of the TB model will aim to include acoustic tests.

**FIG. 5.**
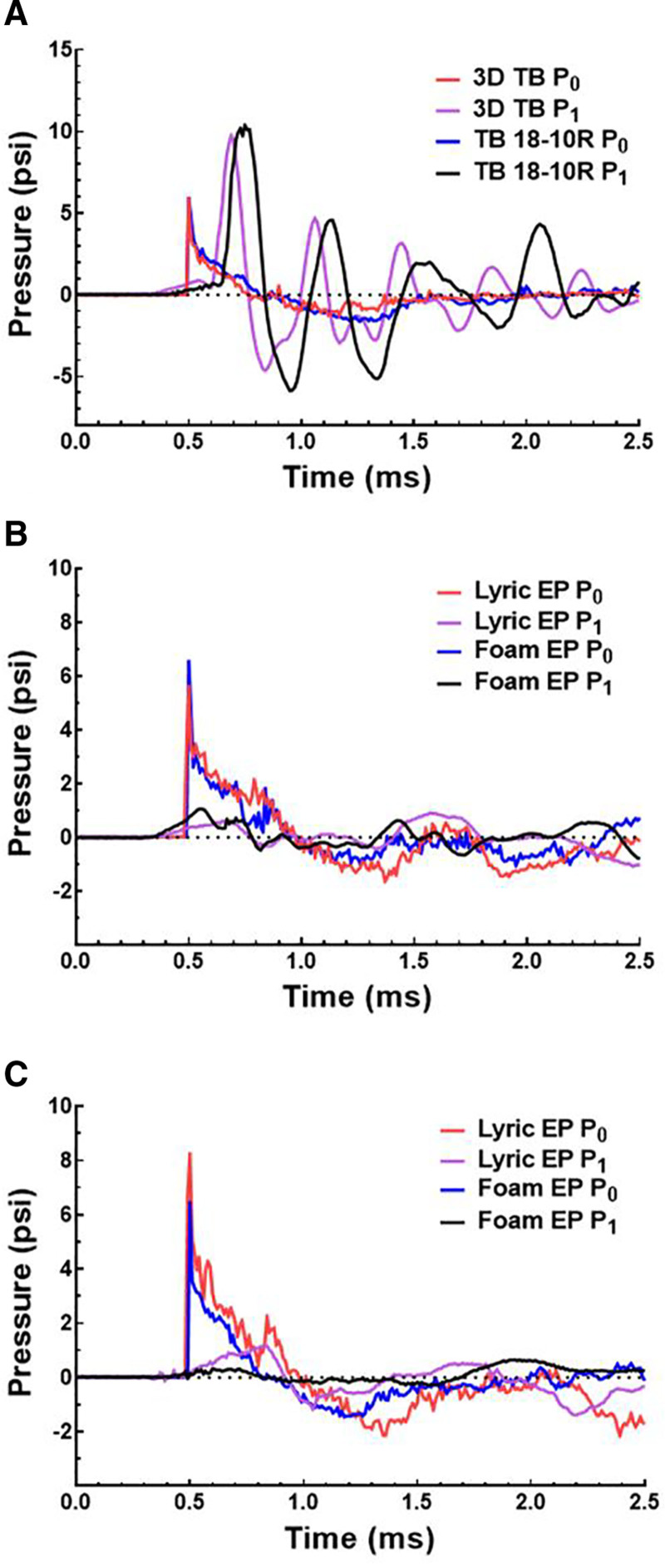
Comparison of pressures measured in human and 3D printed TBs during blast tests with and without EP. *A*, Plot of the P0 (ear canal entrance) and P1 (in the ear canal near the TM) pressures measured in blast tests of the 3D printed TB (red line for P0 and purple line for P1) and a human cadaveric TB (blue line for P0 and black line for P1) without hearing protection. *B* and *C*, Plots of the P0 and P1 pressures measured in blast tests of the 3D printed TB (*B*) and human cadaveric TBs (*C*) protected with a foam EP (blue line for P0 and black line for P1) or Lyric EP (red line for P0 and purple line for P1). EP indicates earplug; TB, temporal bone; TM, tympanic membrane.

The pressures measured at P0 and P1 during blast exposure without an HPD for the 3D printed TB and human TB are shown in Figure 5A. Pressure waveforms in Figure 5 are representative samples with similar P0 peak pressures for detailed comparison. Mean and SDs of blast tests performed with the 3D printed TB are summarized in Table 1. The peak P0 pressure values were 5.89 psi (186 dB) and 5.92 psi (186 dB) for the human TB and 3D printed TB, respectively, and the A-duration of the P0 waveforms for the human TB and 3D printed TB were 0.32 and 0.28 ms, respectively. The P0 waveforms for both test TBs in Figure 5A exhibited very similar magnitudes and shapes. The resulting P1 pressure waveforms had peak values of 10.38 psi (191 dB) and 9.79 psi (191 dB) (5.7% error), which occurred at 0.79 and 0.69 ms (12.7% error) for the human TB and 3D printed TB, respectively. The A-duration of the P1 waveforms were 0.05 and 0.08 ms for the human TB and 3D printed TB, respectively, with a small difference of 0.03 ms. While the P1 peak pressures and A-durations were similar, the time-of-arrival for P1 peak was sooner for the 3D printed TB than with the human TB (Fig. 5A). The P1:P0 ratio was 1.76 for the human TB sample and 1.65 for the 3D printed TB with a relatively low percent error of 6.19%, and the model’s ratio was within the SD reported by Gan et al (9) in human TBs (typically a mean and SD of 1.7 ± 0.5; Table 1).

**TABLE 1.** Summary of the pressure intensities measured during blast exposure tests

TB model (Fig. 5)	P0 (dB) w/out EP	P1 (dB) w/out EP	P1:P0 ratio w/out EP	P0 w/EP (dB)		P1 w/EP (dB)		P0–P1 w/EP (dB)	
				Foam	Lyric	Foam	Lyric	Foam	Lyric
3D printed	186.2	190.6	1.65	187.1	185.7	171.3	170.0	15.8	15.7
Human	186.2	191.1	1.76	186.9	189.1	167.0	172.2	19.9	16.9
TB model (mean ± SD)	P0 (dB) w/out EP	P1 (dB) w/out EP	P1:P0 ratio w/out EP	P0 w/EP (dB)		P1 w/EP (dB)		P0–P1 w/EP (dB)	
				Foam	Lyric	Foam	Lyric	Foam	Lyric
3D printed	186.2 ± 0.03	191.3 ± 0.7	1.8 ± 0.1	185.5 ± 1.9	184.3 ± 1.4	171.1 ± 0.1	169.4 ± 1.1	14.3 ± 1.8	14.9 ± 0.6
Gan et al (9)	^*a*^	194.0 ± 3.1	1.7 ± 0.5	189.1 ± 1.7	189.1 ± 1.3	176.8 ± 7.2	172.8 ± 2.1	12.3 ± 6.4	16.3 ± 1.7

^*a*^Data not available for calculation.

EP indicates earplug; Foam, foam earplug; Lyric, lyric earplug; TB, temporal bone; w, with; w/out, without.

Figure 5B, C show the measured pressures during blast exposure for the human and 3D printed TBs with earplugs equipped. The P0 and P1 pressures recorded while the 3D printed TB had either the foam or Lyric earplug inserted into the ear canal can be seen in Figure 5B, and Figure 5C plots the similar data for that in a human TB. For the 3D printed TB, the peak pressures were 6.54 psi (187 dB) and 1.06 psi (171 dB) for the foam earplug (blue and black lines) and 5.62 psi (186 dB) and 0.92 psi (170 dB) for the Lyric earplug (red and purple lines) at P0 and P1, respectively. The P1 peak times occurred at 0.55 ms and 1.57 ms for the foam and Lyric earplugs, respectively. As for the human TB, the peak pressures were 6.43 psi and 0.65 psi for the foam earplug and 8.24 psi (189 dB) and 1.18 psi (172 dB) for the Lyric earplug at P0 and P1, respectively (Fig. 5C). The P1 peak times occurred at 1.93 ms with the foam earplug and 0.83 ms with the Lyric earplug. Qualitative observation of the results initially revealed that HPDs in the 3D printed TB attenuated blast pressures were like that in human TBs; however, P1 peak pressures and times for the 3D printed TB were 20% greater for all values. Earplug fit and ear canal variation may account for much of the difference between the human and 3D printed TBs. An ear canal designed to better fit standard-sized earplugs can be considered for future model iterations.

The 3D printed TB’s ability to evaluate HPDs’ protective function was within the desired operation range. For the foam earplug, the drop in pressure from the entrance of the ear canal to the TM (P0–P1, in decibels) was 19.9 dB in the human TB and 15.8 dB in the 3D printed TB (20.7% error), and the decibel loss for tests performed in the 3D printed TB was within the SD for tests performed in human cadaveric TBs (9) (mean and SD of 12.3 ± 6.4 dB for foam earplugs). The P0–P1 for the Lyric earplug in the human TB was 16.9 dB and 15.7 dB in the 3D printed TB (7.0% error), and the pressure level drop across the Lyric earplug was also within the reported SD (9) (mean and SD of 16.3 ± 1.7 dB). The Lyric earplug was fitted in both the human and 3D printed TB by an experienced audiologist from Phonak to ensure a correct fit, which may account for the decreased variance among samples.

Table 1 summarizes pressure measurements previously mentioned (in decibels) for Figure 5 and compares the mean and SDs to reported data from Gan et al (9). It can be seen in Table 1 that the data recorded with the 3D printed TB resembles that reported by Gan et al (9), particularly, the P1:P0 ratio (1.8 ± 0.1) and P0–P1 values (14.3 ± 1.8 dB for foam and 14.9 ± 0.6 dB for Lyric, respectively) were within the range of results measured in human cadaveric TB models.

## DISCUSSION

### Application of 3D Printed TB for Evaluation of HPDs

The use of a standardized blast test model for assessing the protective function of HPD designs should greatly assist in HPD development by offering an efficient, accurate, and cost-effective model. With its low material cost, the 3D printed model can be accessible to researchers for less than $100 of material costs while a pair of human donor TBs can cost $1000+. Improved HPDs would undoubtedly reduce the occurrence of hearing loss among veterans (1). Previous publications have reported studies that thoroughly test various HPDs at the SPL of military firearms (7,11) (about 130–170 dB), but few studies investigated the effectiveness of HPD in blast pressures above 180 dB (9,10,22). Gan et al (9) published the most comparable results to this study with blast exposures performed at levels above 185 dB. The P1 pressures measured in the 3D printed TB were similar to that observed by Gan et al (9), and many of the results from the 3D printed TB followed the same trend and were within the SDs by Gan et al (9). In addition, the P1 reduction (P1 with an earplug subtracted from P1 without an earplug) of the foam and Lyric earplugs in the 3D printed TB were 19.3 and 20.5 dB, respectively, which were within the reported SDs (17.3 ± 6.8 dB and 20.9 ± 5.3 dB for the foam and Lyric earplugs, respectively [9]). Brungart et al (22) did report an impulse peak insertion loss (ie, P0–P1) of 30–34 dB at blast pressures from 150 to 190 dB using a Lyric extended wear hearing aid. The greater than 10 dB difference in attenuation may be due to the use of an acoustic test fixture and the Lyric hearing aid needing to be placed against the fixture’s microphone (ie, eardrum) due to the short length of its ear canal. The Lyric hearing aid was inserted at the correct length with our anatomically accurate 3D printed TB. The 3D printed TB, with HPD attenuation function measurements similar to literature (9,10,22), demonstrated that this TB model may function as an accurate standardized tool for efficiently evaluating passive HPDs’ performance.

### Limitations and Future Work

The middle ear tissues attached to ossicular chain are viscoelastic in which their response to stimuli is heavily dependent on time or strain rate; however, the materials used to print the TB exhibited an elastic behavior. The LDV displacement differences between the 3D printed and human TMs should be due to the lack of viscoelastic behavior of the 3D printed TM and middle ear. A 3D printed TB that can model the mechanical response of the ear from blast and acoustic stimuli would be greatly beneficial to the development of HPDs. Refinement of the acoustic response of the 3D printed TB’s middle ear is planned for future iterations of our model.

Moreover, discrepancies between the LDV data from the 3D printed and human TBs (Fig. 4) indicated that the 3D printed TB’s middle ear transfer function needs to improve (24). To address the middle ear’s mass-damping balance, the addition of a cochlear load will further improve the acoustic response of our 3D printed TB. As shown in Figure 1D and Figure 5B, the design of the 3D printed TB considered this to provide the fluid pressure feedback from the cochlea during ossicular movement. Once the middle ear transfer function of the 3D printed TB is improved, LDV measurements of the TM and ossicular chain are planned to further validate our model.

## CONCLUSIONS

In summary, a 3D printed TB was created for the purpose of providing standardized testing of HPDs to blast exposure. Two HPDs (standard foam earplug and Lyric hearing aid) were included for testing the protective function with the 3D printed TB during blast. The attenuated peak pressure near the TM was as low as 0.92 psi (170 dB) with a blast peak pressure of 5.62 psi (186 dB) at the entrance of the ear canal with a HPD in use, and without a HPD, the pressure near the TM was 9.79 psi (191 dB) with a similar blast peak at the entrance of the ear canal. Results show that the pressure measurements in the 3D printed TB were well within the mean and SD of the published data from tests performed in human cadaveric TBs demonstrating that our 3D printed TB is a valid model for testing passive HPD designs. In this study, the 3D printed TB was developed for evaluating HPDs’ protective function against blast overpressure exposure and performed comparable to the human cadaveric TB model providing an accessible and cost-effective alternative tool.

## FUNDING SOURCES

The study was supported by the Department of Defense (DOD) grant W81XWH-14-1-0228.

## CONFLICT OF INTEREST

None declared.

## DATA AVAILABILITY STATEMENT

The datasets generated during and/or analyzed during the current study are not available.

## Supplementary Material


