# Supplementary material for: A 3D Printed Human Ear Model for Standardized Testing of Hearing Protection Devices to Blast Exposure
Source: Otol Neurotol Open. 2022 May 26;2(2):e010. doi: 10.1097/ONO.0000000000000010 (PMC10950174; doi:10.1097/ONO.0000000000000010)
Supplement: Supplementary file 1 [file ono-2-e010-s001.pdf]

SUPPLEMENTAL DIGITAL CONTENT 1

Table S1. Material properties of the 3D printed materials used for each part in the 3D printed TB.

Structure	3D Printer Material Properties[a]		
	**Tensile Strength (MPa)**	**Elong. at Break (%)**	**Young's Mod. (MPa)**
Tympanic Membrane	1.3 - 1.8	125%	
Skin	3.5 - 5.0	75%	
Manubrium	40 - 60	25%	1700 - 2300
Ossicles & TB	50 - 65	10 - 25%	2000 - 3000
Stapedial Annular Ligament	0.8 - 1.5	170 - 220%	
Other Ligaments	1.3 - 1.8	125%	
Tympanic Annulus	1.3 - 1.8	125%	
Posterior Stapedial Tendon	3.5 - 5.0	75%	
Tensor Tympani Tendon	1.9 - 3.0	105%	
Incudostapedial Joint	2.5 - 4.0	80%	
Incudomallear Joint	8.5-10.0	50%	

Abbreviations: Elong. = Elongation; Mod. = Modulus; TB = Temporal Bone

[a] *Materials used in the Objet350 are proprietary and material properties are reported as published by Stratasys Ltd.* [20,21]
